# Account of Diseases in an Eastern District of London, from the 20th of October to the 20th of November, 1801

**Published:** 1801-12

**Authors:** 


					[ 490 ]
Account of Diseases in an'Eastern District of London, from the
20th of October to. the 20th of November, 1801.
ACUTE DISEASES.
Scarlatina Anginofa - - 2
Typhus - -- -- -12
Peripneumonia - - - 3
Eryfipelas - - - - - 1
Dyfenteria ----- 5
Cholera Morbus - - - 3
CHRONIC DISEASES.
Peripneumonia Notha - - 6
T ufiis ------ 16
Dyfpncea ----- 13
Hoemoptoe ----- 4
Phthifis Pulmonaljs - - 3
Hydrothorax - - - - 2
A (cites ------ 3
Anafarca - - - - - 4
Cephalalgia ----- 5
Vertigo ------ 3
Paralyfis - - - - - 2
Haemorrhois - - - - 3
Amenorrhea - - - - 6
Chlorofis ----- 5
Leucorrhcea - - - - 4
Diarrhoea - - - - - 2
Vomitus ------ i
Dyfuria ------ 3
Herpes ------ 7
Rheumatifmus Chronicus 14.
PUERPERAL DISEASES.
Ephemera ----- 3
Menorrhagia Lochialis - 2
Abfpeflus Mamma; - - - I
INFANTILE DISEASES.
Aphthse ------ 3
Herpetic Eruptions - - 4
^ Hooping Cough - - - z
Diarrhoea - - ? - - 4
The cafes of Typhus will appear to have decreafed in num-
ber fince the Iaft Report. , We are happy to announce that
this fpecies of fever, which has fo long prevailed, and the in-
fluence of which has been fo extenlively diffufed, has lately
appeared in much fewer cafes, and that thefe have not been
attended with fo many fevere fymptoms as have formerly en-
gaged our attention. Peripneumonies, catarrhs, and other af-
fections of the refpiratory organs, form, as they ufually do at
this period of the year, a confiderable part of the lift of dif-
eafes. Difeafes of the bowels in different forms {till continue.
One of the cafes of dyfentery referred to in the Lift, was at-
tended with fymptoms not ufually connected with this difeafe.
The patient, who was much advanced in life, after labouring
for fome time under a fevere dyfentery, on rifing one morning
obferved a foot of a livid appearance on one of her arms^
which was foon fucceeded by others j and, in a ftiort time,
vibices appeared in almoft every part of the body. An un-
pleafant fenfation in her mouth and gums was followed by. a
confiderable difcharge of blood from them, by which the whole
furface of the internal fauces was tinged. During the pre-
fence of thefe fymptoms, the dyfenteric affe?tions were fuch as
to require immediate attention, and to forbid the ufe of remer
dies more directly adapted to the removal of the other difeafe.
By the ufe of pulv- ipec. comp. followed by the exhibition of
briik
brifk cathartics, the dyfenteric fymptoms were relieved, the
ejection of fasces was procured, and the pain of the bowels
ceafed. Afterwards, in the free ufe of bark and acids, the
vibices gradually loft their livid hue, and in the courfe of a few
days difappeared.
+ 1 .J ? J. .

				

